# The Role of P-Glycoprotein in Transport of Danshensu across the Blood-Brain Barrier

**DOI:** 10.1155/2011/713523

**Published:** 2011-01-20

**Authors:** Peng-Fei Yu, Wen-Yan Wang, Gaowa Eerdun, Tian Wang, Lei-Ming Zhang, Chong Li, Feng-Hua Fu

**Affiliations:** ^1^Department of Pharmacology, School of Pharmacy, Yantai University, Yantai, Shandong 264005, China; ^2^The First Hospital of Hebei Medical University, Shijiazhuang, Hebei 050031, China

## Abstract

Danshensu (3-(3, 4-dihydroxyphenyl) lactic acid), a water-soluble active component isolated from the root of *Salvia miltiorrhiza* Bunge, is widely used for the treatment of cerebrovascular diseases. The present study aims to investigate the role of P-glycoprotein in transport of Danshensu across the blood-brain barrier. Sprague-Dawley rats were pretreated with verapamil at a dose of 20 mg kg^−1^ (verapamil group) or the same volume of normal saline (control group). Ninety minutes later, the animals were administrated with Danshensu (15 mg kg^−1^) by intravenous injection. At 15 min, 30 min, and 60 min after Danshensu administration, the levels of Danshensu in the blood and brain were detected by high-performance liquid chromatography-electrospray ionization tandem mass spectrometry (LC-ESI-MS/MS). The results showed that Danshensu concentrations in the brain of the rats pretreated with verapamil were significantly increased. In addition, the brain-plasma ratios of the group pretreated with verapamil were much higher than that of the control group. There was no difference in Danshensu level in plasma between the verapamil group and control group. The findings indicated that Danshensu can pass the blood-brain barrier, and P-glycoprotein plays an important role in Danshensu transportation in brain.

## 1. Introduction

The blood-brain barrier (BBB) is a diffusion barrier, consisting of an interdependent network of cells designed to segregate the central nervous system (CNS) from the systemic circulation. BBB can prevent the movement of drugs from the blood into the brain, and therefore acts as an obstacle for the systemic delivery of neurotherapeutics. Furthermore, efflux transport systems may target the drugs and export them from the brain, in which P-glycoprotein (P-gp) plays a crucial role.

P-gp is a member of the ATP-binding cassette superfamily of transmembrane transporters which mediates the membrane transport of many hydrophobic compounds, including hormones, sterols, lipids, phospholipids, cytokines, and anticancer drugs [1]. P-gp is located in many tissues and in the capillary endothelial cells of the testis and the BBB, where it functions as an efflux transporter of xenobiotics. Interactions with substances that inhibit P-gp are of great interest, as they can potentially enhance the absorption of important medicines that are generally poorly absorbed, such as drugs for CNS. Verapamil is the most extensively characterized P-gp inhibitor and multidrug resistance-associated protein (MDR) reversal agent [2]. It is also reported that coadministration of verapamil with a recognized antidepressant (imipramine) improves the clinical outcome in previously resistant cases and the inhibition of P-gp was a potential mechanism of action for verapamil during treatment resistant depression [3]. 

Danshen, the dried root of *Salvia miltiorrhiza* Bunge, is widely used for the treatment of various microcirculatory disturbance-related diseases, such as cardiovascular disease, liver dysfunction, and cerebrovascular disease [4]. Chemical constituents of *Salvia miltiorrhiza* Bunge are classified into two major categories: lipophilic compounds (such as Tanshinone) and hydrophilic compounds (such as Danshensu). Studies have showed that lipophilic compounds Tanshinone I, Tanshinone IIA, Cryptotanshinone, and 15, 16-dihydrotanshinone I had the ability to ameliorate memory deficits induced by scopolamine [5]; Tanshinone IIA and Tanshinone IIB could lead to reduction of brain infarct volume and the restoration of neurological function in an experimental model of stroke in mice [6]; Cryptotanshinone could improve the cognitive ability in Alzheimer's disease transgenic mice [7]. Besides, Tanshinone I [8], Tanshinone IIA [9], and Cryptotanshinone [10] were also found to be the substrates of P-gp. However, it is still unclear whether Danshensu, a hydrophilic compound in Danshen, has the potential of crossing the BBB or is the substrate of P-gp. The present study aims to investigate the role of P-gp in the transport of Danshensu across the BBB by observing Danshensu concentration in plasma and brain tissue in rats.

## 2. Material and Methods

### 2.1. Drugs and Chemicals

Danshensu (Purity 99.6%) was obtained from Shandong Luye Pharmaceutical Co., Ltd. (Yantai, China). Verapamil was obtained from Shanghai Hefeng Pharmaceutical Co., Ltd. (Shanghai, China). Naproxen (Purity 99.9%) was obtained from National Institute for the Control of Pharmaceutical and Biological Products. Ethyl acetate (analytical reagent) was obtained from Sinopharm Chemical Reagent Co., Ltd. (Shanghai, China). Acetonitrile (Purity 99.9%) was obtained from Merck (Darmstadt, Germany).

### 2.2. Animals and Treatment

Forty-eight male Sprague Dawley rats weighing 220 ± 20 g were provided by the Experimental Animal Center of Shandong Engineering Research Center for Natural Drugs (Yantai, China), certificate number 20030020. All experimental procedures carried out in this study were performed in accordance with the guidelines for the Care and Use of Laboratory Animals of Yantai University. The rats were kept with free access to food and water on a 12 h light/dark cycle. They were housed in plastic cages and randomly divided into two groups with 24 animals in each group: the control group and the verapamil group. The rats in the verapamil group were administered intraperitoneally with verapamil at a dose of 20 mg kg^−1^. The rats in the control group were treated with the same volume of normal saline. Ninety minutes later, all rats were treated intravenously with Danshensu (15 mg kg^−1^, i.v.) by tail vein. At 15 min, 30 min, and 60 min after Danshensu treatment, the animals were anesthetized with chloral hydrate (300 mg kg^−1^, i.p.) and then 5 mL heparinized blood (1% heparin, 100 *μ*L) were collected from abdominal aorta and the rats were perfused with 100 mL of ice-cold normal saline each. The brain was rapidly removed from the cranium and weighed. Then the brain was homogenized in 4 volumes of 0.1 mol L^−1^ ice phosphate buffer (pH 7.4). Three milliliters of ethyl acetate was added into 200 *μ*L of the homogenate. After vortexing for 3 min and centrifuging (2500 g, 4°C) for 5 min, the supernatants were evaporated to dryness under a gentle nitrogen stream at 40°C. The residues were resuspended in mobile phase (the composition will be referred in the following text). The blood samples were centrifugated (2500 g, 4°C) for 10 min and plasma was separated. Plasma was treated as described for brain homogenate supernatants.

### 2.3. Chromatographic Conditions

The chromatographic separation was performed using an Agilent 1100 Series HPLC system equipped with a vacuum degasser, a quaternary pump, an autosampler, and a column oven. The chromatographic separation was run on a Hanbon ODS-C18 column (150 × 2.1 mm, 3 *μ*m). The mobile phase was acetonitrile-water (55 : 45, v/v). The pump was operated at a flow rate of 0.2 mL min^−1^. Separations were performed at the temperature of 20°C.

### 2.4. Mass Spectrum Conditions

Mass spectrometric detection was performed using a TSQ Quantum tandem mass spectrometer equipped with an electrospray ionization (ESI) source (Thermo Electron Corporation, USA). Quantification was performed using selected reaction monitoring (SRM) of the transitions of *m/z* 197.0 → *m/z *135.1 for Danshensu and *m/z *229.0 → *m/z* 170.1 for the naproxen (Internal Standard). The mass spectrum conditions were optimized as follows: spray voltage, 3000 V; sheath gas pressure, 30 psi; auxiliary gas pressure, 5 arbitrary unit; capillary temperature, 350°C; collision-induced dissociation voltage, 18 V; argon gas pressure, 1.5 millitorr. Data acquisition was performed with Xcalibur software.

Ionization was operated in negative Selected Ion Monitoring (SIM) mode. Sheath gas (N_2_) pressure was 30 kPa and aux gas (N_2_) pressure was 5 kPa. Capillary temperature was 150°C. Ion sweep gas pressure was 0 kPa and Tube Lens offset was 105 eV.

### 2.5. Statistical Analysis

Data is expressed as means ± SEM. The statistical significances of the data were determined using one-way analysis of variance (ANOVA) followed by the Least Significant Difference testing. The *P* value < .05 was considered as statistically significant.

## 3. Results

### 3.1. High-Performance Liquid Chromatogram of Danshensu

**Figure 1:**
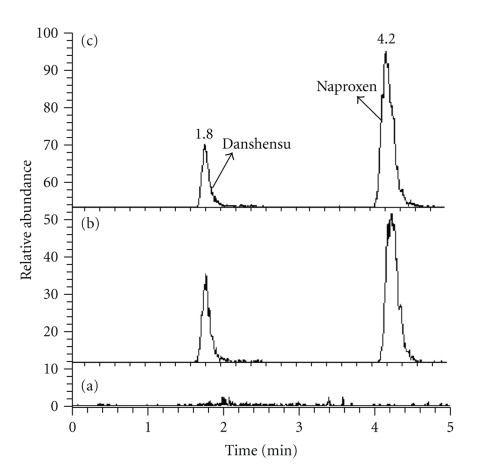
SRM chromatograms of Danshensu and naproxen (Internal Standard) in blank rat brain (a), blank brain sample spiked with Danshensu (Standard) and naproxen (b) and Danshensu treated rat brain spike with naproxen (c).

**Figure 2:**
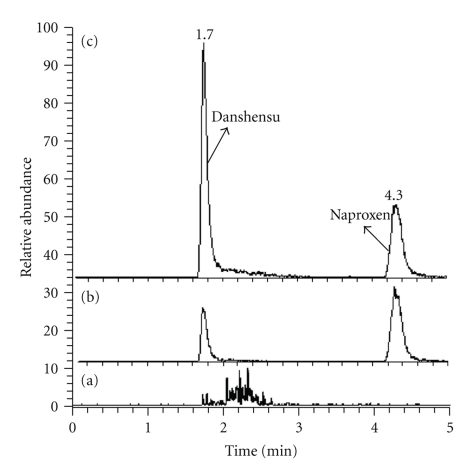
SRM chromatograms of Danshensu and naproxen in blank rat plasma (a), blank plasma sample spiked with Danshensu and naproxen (b) and Danshensu treated rat plasma spike with naproxen (c).

Figures 1 and 2 show the typical SRM chromatograms of the blank rat brain; brain spiked with Danshensu (Standard) and naproxen (Internal Standard); brain of Danshensu treated rat with spike of naproxen (Internal Standard); blank rat plasma; plasma spiked with Danshensu and naproxen; plasma of Danshensu treated rat with spike of naproxen. The retention times of Danshensu and naproxen were 1.8 and 4.2 min in brain and 1.7 and 4.3 min in plasma, respectively.

### 3.2. Effect of Verapamil on Danshensu Concentrations in Brain

**Figure 3:**
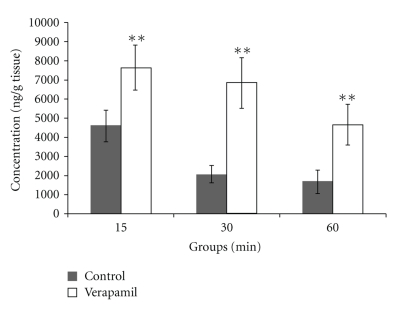
Effect of verapamil on Danshensu concentration in rat brain. Data was expressed as mean ± SEM (*n* = 8). Statistical significances were determined using one-way analysis of variance (ANOVA) followed by the Least Significant Difference testing. **P < .01 compared with control group.

At 15 min, 30 min, and 60 min after Danshensu treatment, Danshensu concentrations in the brain of the verapamil group were significantly higher than that of the control group (1.67, 3.32 and 2.80 fold, resp., *P* <  .01, Figure 3). 

### 3.3. Effect of Verapamil on Danshensu Concentrations in Plasma

**Figure 4:**
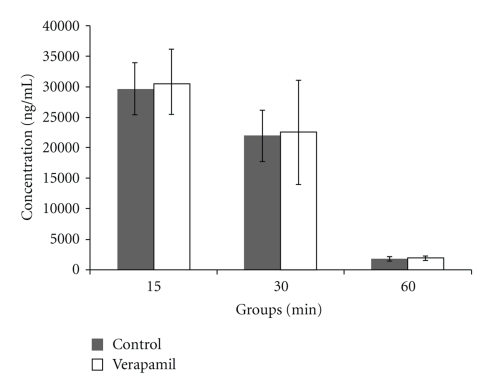
Effect of verapamil on Danshensu concentration in rat plasma. Data was expressed as mean ± SEM (*n* = 8). Statistical significances were determined using one-way analysis of variance (ANOVA) followed by the Least Significant Difference testing.

Compared with control, pretreatment with verapamil had no effect on Danshensu concentrations in plasma (Figure 4).

### 3.4. Effect of Verapamil on Danshensu Concentrations in Brain : Plasma Ratios

**Figure 5:**
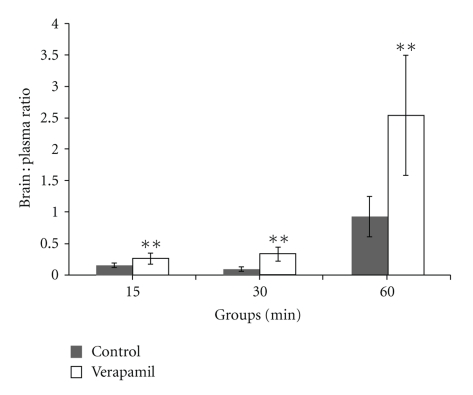
Effect of verapamil on brain:plasma ratio of Danshensu concentration in rats. Data was expressed as mean ± SEM (*n* = 8). Statistical significances were determined using one-way analysis of variance (ANOVA) followed by the Least Significant Difference testing. **P < .01 compared with control group.

At 15 min, 30 min, and 60 min after Danshensu treatment, the brain-plasma ratios in Danshensu concentrations of verapamil group were significantly increased (*P* < .01, Figure 5).

## 4. Discussion

BBB, being made up of the brain capillary endothelial cells which are connected to each other by well-developed tight junctions, is a lipoid membrane barrier. Because of its strict regulation on the movement of compounds from the circulating blood into the brain, permeation of xenobiotics across the BBB has long been believed to be dependent on their lipophilicity. However, increasing studies reported that the permeation of the highly lipophilic drugs, for example, vinca alkaloid, doxorubicin, and cyclosporin A, across the BBB is unexpectedly low. Studies on the BBB transport of xenobiotics, as well as nutrients and neuroactive agents, have led to a change in the concept of the BBB. BBB is no longer regarded as a static lipoid membrane barrier of endothelial cells, but rather is considered to be a dynamic interface that has physiological functions for the specific and selective transmembrane transport of many compounds [11].

The apparently contradictory observations can be ascribed to the existence of multiple mechanisms of drug transport through the BBB. The MDR1 gene product P-gp is a membrane protein, which functions as an ATP-dependent exporter of xenobiotics from cells. P-gp is expressed in normal tissues with excretory functions such as the intestine, liver, kidneys, and capillary endothelial cells of the brain. Several studies pointed to a predominant role of the efflux transporter P-gp as a major gatekeeper in the BBB [12, 13]. P-gp has a profound effect on the entry of drugs, peptides and other substances into the CNS. High level of expression, multispecificity, and high transport potency makes P-gp as a primary obstacle to drug delivery into the brain, thereby contributing to the poor success rate of a large range of therapeutic candidates, and probably contributing to patient-to-patient variability in response to CNS pharmacotherapy. Although it reported that Danshensu had a protective effect against experimental impairment of memory induced by cerebral ischemia reperfusion [14], it remains unclear whether Danshensu could cross BBB.

Our results demonstrated that at 15 min after Danshensu administration, its concentration in the brain reached a relatively high level in both the control and verapamil groups, which indicates that Danshensu can cross the BBB. Furthermore, the concentration of Danshensu in the verapamil group was much higher than that of control, but verapamil did not affect the concentration of Danshensu in plasma, which suggested that the effect of verapamil on the concentration of Danshensu in the brain did not depend on the interfering of the elimination of Danshensu from blood. In turn, it may be deduced that P-gp played an important role in effluxion of Danshensu from the brain because verapamil, as an inhibitor of P-gp, could increase the concentration of Danshensu in the brain.

It should be noted that the present experiment only evaluated the role of P-gp which played on Danshensu. However, the effect of Danshensu on P-gp expression has not been taken into consideration. As a result, our further studies will focus on whether Danshensu could modulate the function or expression of P-gp.

 In summary, the present study demonstrated that Danshensu can pass BBB. It was also indicated that inhibiting P-gp could therefore increase the concentration of Danshensu in brain. Subsequently, our studies highlight the importance of P-gp inhibitor as a coadministration with Danshensu in the therapy of CNS disorders.
